# MutMap Approach Enables Rapid Identification of Candidate Genes and Development of Markers Associated With Early Flowering and Enhanced Seed Size in Chickpea (*Cicer arietinum* L.)

**DOI:** 10.3389/fpls.2021.688694

**Published:** 2021-07-12

**Authors:** Praveen Kumar Manchikatla, Danamma Kalavikatte, Bingi Pujari Mallikarjuna, Ramesh Palakurthi, Aamir W. Khan, Uday Chand Jha, Prasad Bajaj, Prashant Singam, Annapurna Chitikineni, Rajeev K. Varshney, Mahendar Thudi

**Affiliations:** ^1^Center of Excellence in Genomics and Systems Biology (CEGSB), International Crops Research Institute for the Semi-Arid Tropics (ICRISAT), Hyderabad, India; ^2^Department of Genetics, Osmania University, Hyderabad, India; ^3^Indian Council of Agricultural Research (ICAR)-Indian Agricultural Research Institute, Regional Research Centre, Dharwad, India; ^4^Indian Council of Agricultural Research (ICAR)-Indian Institute of Pulses Research (IIPR), Kanpur, India; ^5^State Agricultural Biotechnology Centre, Centre for Crop and Food Innovation, Food Futures Institute, Murdoch University, Murdoch, WA, Australia; ^6^Centre for Crop Health, University of Southern Queensland, Toowoomba, QLD, Australia

**Keywords:** MutMap, early flowering, chickpea, 100 seed weight, candidate genes and SNPs

## Abstract

Globally terminal drought is one of the major constraints to chickpea (*Cicer arietinum* L.) production. Early flowering genotypes escape terminal drought, and the increase in seed size compensates for yield losses arising from terminal drought. A MutMap population for early flowering and large seed size was developed by crossing the mutant line ICC4958-M3-2828 with wild-type ICC 4958. Based on the phenotyping of MutMap population, extreme bulks for days to flowering and 100-seed weight were sequenced using Hi-Seq2500 at 10X coverage. On aligning 47.41 million filtered reads to the CDC Frontier reference genome, 31.41 million reads were mapped and 332,395 single nucleotide polymorphisms (SNPs) were called. A reference genome assembly for ICC 4958 was developed replacing these SNPs in particular positions of the CDC Frontier genome. SNPs specific for each mutant bulk ranged from 3,993 to 5,771. We report a single unique genomic region on Ca6 (between 9.76 and 12.96 Mb) harboring 31, 22, 17, and 32 SNPs with a peak of SNP index = 1 for low bulk for flowering time, high bulk for flowering time, high bulk for 100-seed weight, and low bulk for 100-seed weight, respectively. Among these, 22 SNPs are present in 20 candidate genes and had a moderate allelic impact on the genes. Two markers, Ca6EF10509893 for early flowering and Ca6HSDW10099486 for 100-seed weight, were developed and validated using the candidate SNPs. Thus, the associated genes, candidate SNPs, and markers developed in this study are useful for breeding chickpea varieties that mitigate yield losses under drought stress.

## Introduction

Chickpea (*Cicer arietinum* L.) is the second most important annual grain legume crop predominantly cultivated on residual soil moisture in the arid and semi-arid areas of the world. Global annual cultivation of chickpea is over 14.56 million ha with a total production of 14.77 million tons (FAOSTAT, 2017, accessed on January 26, 2020). Chickpea seeds are rich in protein (17–20%), minerals (phosphorus, calcium, magnesium, iron, and zinc) (Jukanti et al., 2012; Sab et al., 2020), and carotenoids; chickpea also improves soil health by adding atmospheric nitrogen (20–40 kg N ha^–1^) through symbiosis (Joshi et al., 2001). Climate changes during the recent past have been posing serious threats to chickpea production and causing about 19% yield losses (Kadiyala et al., 2016).

In India, chickpea is grown in a wide range of agro-climatic niches. Based on crop duration, these regions can be classified as short-duration (Southern/peninsular India), medium-duration (Central India), and long-duration (Northern India) environments. In general, chickpea matures in a wide time frame of 80–180 days. However, in 66% of chickpea-growing areas, the available crop-growing season is about 80–120 days as they are exposed to abiotic stresses such as drought and heat toward the grain-filling stage. A major shift in the chickpea area (about 3 million ha) from Northern India (cooler, long-season environment) to Southern India (warmer, short-season environment) has been observed during the past four decades. As a result, no major boost in the total production of chickpea has been substantiated.

Terminal drought is considered as one of the most important constraints to chickpea production, and almost 40–50% yield losses were observed globally (see Roorkiwal et al., 2020). The number of days to flowering is an important trait for crop adaptation and productivity, especially in arid and semi-arid regions that experience terminal drought conditions. Early phenology, an adaptation-related trait, helps in the adaptation of chickpea to short-season environments as early flowering genotypes escape terminal (end of season) stresses (drought, high/low temperature) (see Berger et al., 2006). Therefore, the ability to manipulate flowering time is an essential component of chickpea improvement. Seed size/weight is an important yield-contributing trait, and therefore, in past, major breeding emphasis was on improving this trait (Gaur et al., 2014). As a result, early flowering desi and kabuli genotypes were identified through germplasm characterization (Upadhyaya et al., 2007); the low-resolution quantitative trait loci (QTLs) have been reported for flowering time and seed size (Varshney et al., 2014c; Upadhyaya et al., 2015; Verma et al., 2015; Mallikarjuna et al., 2017). Further, efforts were also made to understand the genes and pathways involved in flower development in chickpea (Singh et al., 2013), including through a gene expression atlas (Kudapa et al., 2018). Although the QTLs mapped within large genomic intervals limit the identification of potential candidate genes and their use in marker-assisted selection, in recent years, using a marker-assisted backcrossing approach several high-yielding and drought-tolerant lines in different genetic backgrounds of chickpea have been released for cultivation (Varshney et al., 2013a; Bharadwaj et al., 2021). Molecular breeding lines with enhanced resistance to biotic stresses were also developed (Varshney et al., 2014b; Pratap et al., 2017; Mannur et al., 2019).

The majority of the QTL mapping and gene isolation approaches using traditional approaches are time-consuming and low-throughput methods. Nevertheless, for more than a decade, the next-generation sequencing (NGS) technologies facilitated understanding of the genetics of complex traits at a faster pace in cereals and legumes (Thudi et al., 2020; Jaganathan et al., 2020). In the case of chickpea, apart from sequencing the genome (Varshney et al., 2013b) and several hundred germplasm lines (Thudi et al., 2016a, b; Varshney et al., 2019b), traits were fine-mapped (Kale et al., 2015; Singh et al., 2016). For decades, forward genetic approaches that rely on molecular characterization of altered phenotypes have been one of the driving forces for crop improvement. In the case of crops with a narrow genetic base, such as chickpea, the creation of allelic variation through mutations and the identification of causal variants will be a potential alternative that can overcome the existing production barriers. MutMap is one of the novel gene mapping approaches that allows rapid identification of causal nucleotide changes of mutants by whole-genome resequencing of pooled DNA of mutant F_2_ progeny derived from crosses made between candidate mutants and the parental line (Abe et al., 2012; Fekih et al., 2013). This new NGS-based technique has been successfully applied in crop plants for rapid identification of the candidate gene as well as the QTL responsible for agronomically important traits (Abe et al., 2012; Megersa et al., 2015; Takagi et al., 2015; Fang et al., 2016; Klein et al., 2018; Tran et al., 2020).

Here, we report for the first time in chickpea, deployment of the MutMap approach that enabled us to rapidly identify genes and single nucleotide polymorphisms (SNPs) associated with early flowering and seed size. In addition, we also report the development and validation of markers that can be used for selection in chickpea breeding programs for improving these traits.

## Materials and Methods

### Development and Phenotyping of MutMap Population

To identify phenotypically distinct mutant lines for early flowering and larger seed size, a set of 100 mutant lines from a TILLING (target-induced local lesions in the genome) population developed through ethyl methanesulfonate (EMS) mutagenesis of desi chickpea genotype ICC 4958 (unpublished ICRISAT) was phenotyped for these traits at ICRISAT (17.5111° N, 78.2752° E).

ICC 4958 is a drought-tolerant accession available from the ICRISAT germplasm collection. It was collected from Jabalpur, Madhya Pradesh, India, in 1973, and it was among the over 1,500 germplasm accessions screened for drought resistance at ICRISAT Center between 1978 and 1983. It is being used as a donor parent for introgressing drought tolerance-related traits and that produces high yields in low productivity, short-duration, terminal drought-prone environments, e.g., those in peninsular India (Varshney et al., 2013a; Bharadwaj et al., 2021).

A set of 45 simple sequence repeat (SSR) markers distributed equally across the genomes was used to identify the genetic similarity among the selected lines. SSR genotyping was performed as described earlier (Thudi et al., 2011). PCR products were denatured and size-fractioned using capillary electrophoresis on an ABI 3730 DNA Genetic Analyzer (Applied Biosystems, United States). Based on allelic data, the mutant line with > 95% similarity to ICC 4958 was selected as the female parent. A MutMap population was developed crossing ICC 4958-M3-2828 (with large seed size and early flowering) and ICC 4958. F_1s_ were selfed to produce F_2_ seeds. These F_2_ seeds were sown in the field during crop season 2017–2018 at ICRISAT. The F_2_ population was scored for days to flowering (DF) and 100-seed weight (SDW).

### Isolation of DNA and Sequencing of ICC 4958 (Wild Type) and Trait Bulks

Genomic DNA was extracted from the leaves of F_2_ individuals using the NucleoSpin Plant II kit (Macherey-Nagel, Dren, Germany). An equimolar concentration of DNA from 15 F_2_ plants with high phenotypic values was pooled together as high bulk, and similarly, DNA from low phenotypic values was pooled together as low bulk. Thus, four extreme bulks, two for each trait, were prepared for WGRS along with wild-type parent ICC 4958 separately. About 5 μg of the pooled DNA was used for the preparation of a sequencing library of average insert size 200–500 bp, according to the protocol for the Paired-End DNA Sample PrepKit (Illumina, United States). The library was sequenced to 10X of genome coverage with the Illumina HiSeq 2500 platform (Illumina, United States).

### Alignment of Short Reads to Reference Sequences and SNP Calling

Initially, a reference-based sequence of the ICC 4958 wild type was generated by aligning the sequence data generated to the CDC Frontier reference genome (Varshney et al., 2013b) as described in the study by Abe et al. (2012). In brief, 59 million paired-end short reads from ICC 4958 wild type and four mutant pools were used for the analysis. The quality checks for these reads were performed using FastQC v0.11.8 (Andrews, 2010), and Trimmomatic v0.39 (Bolger et al., 2014) was used to filter poor-quality reads and remove potential adapter contamination. For this, Illumina adapters and primers sequences were used by Trimmomatic for trimming, followed by iterative removal throughout the read length with mean base Phred qualities > 30 in 5-bp sliding windows. Remaining sequences with lengths < 35 bp after trimming were discarded as well as orphan single-end reads. These high-quality short reads were pooled and aligned with MAQ to the CDC Frontier reference sequence. Alignment files were converted to SAM or BAM files using SAMtools (Li et al., 2009) and applied to a filter pipeline (Kosugi et al., 2013) for the identification of reliable SNPs. This filter pipeline was developed to maximize true SNP detection and minimize false SNP calling by (i) the removal of paired-end reads of insert size > 325 bp, (ii) calling SNPs only for genomic regions covered by a minimum of three reads for homozygous SNPs and five reads for heterozygous SNPs and a maximum of threefold of average read depth over the genome, and (iii) calling SNPs only on sites with an averaged Illumina Phred-like quality score ≥ 20. Using this pipeline, we identified 332,395 reliable SNPs between ICC 4958 reads and the CDC Frontier reference sequence. On the basis of this result, we generated an ICC 4958 reference sequence by replacing CDC Frontier nucleotides with those of ICC 4958 at 332,395 sites. To remove the effect of SNPs irrelevant to the mutant screen, we generated and used a reference sequence of the same wild-type ICC 4958 that was used for mutagenesis. We further refined this reference sequence by taking a consensus of cumulative genome sequences of the mutants.

Paired-end sequence reads of bulked DNA of mutant F_2_ progeny were aligned to the ICC 4958 reference sequence, and SNPs were scored as homozygous SNPs (with SNP index ≥ 0.9) and heterozygous SNPs (with SNP index ≥ 0.3 and < 0.9). We further excluded common SNPs shared by at least two mutant lines as well as G→A or C→T transitions (as they are most frequent in EMS mutagenesis). After identifying the genomic regions harboring a cluster of SNPs with an SNP index of 1, we relaxed the condition of the filter to consider all SNPs (caused by all the transition and transversion) in the region as candidate SNPs for the causal mutation. SNP index plot regression lines were obtained by averaging SNP indices from a moving window of five consecutive SNPs and shifting the window one SNP at a time. The *x*-axis value of each averaged SNP index was set at a midpoint between the first and the fifth SNP.

### Primer Designing and Validation

The candidate SNPs with a SNP index = 1 were targeted for designing allele-specific markers. WASP, a web-based tool, was used for designing allele-specific primers (Wangkumhang et al., 2007)^1^. A total of 82 desi chickpea genotypes (47 for early flowering and 48 for seed size) were selected for marker validation. PCR was carried out in a 5 μl volume containing 10 ng of DNA, 1X buffer, 200 μM dNTP, 2.5 mM MgCl_2_, 1–5 picomole forward and reverse primers, and 0.1 U of *Taq* polymerase. PCR was performed using Perkin Elmer 384-well Thermal cyclers (Applied Biosystems, United States) and involved a touchdown PCR. Touch down PCR cycles involved initial denaturation at 94°C for 5 min followed by 10 cycles of denaturation at 94°C for 20 s, 60°C for 30 s, 72°C for 30 s that decreases 1°C per cycle; then 35 cycles of 94°C for 20 s, at an optimized annealing temperature of each primer pair (51–58°C) for 30 s, 72°C for 30 s; final extension of 72°C for 20 min, and hold at 4–10°C forever. The PCR products were checked on 1.2% agarose gel containing 0.5 μl/10 ml ethidium bromide (10 mg/ml) with a 100-bp DNA ladder by running it at a constant voltage of 90 V for 25 min. The amplification was visualized under UV illumination using the Uvi-Tech gel documentation system (DOL-008.XD, England).

## Results

### MutMap Population for Early Flowering and Large Seed Size

Based on phenotyping of 100 mutant lines from the TILLING population, 25 lines that were phenotypically distinct from the wild-type ICC 4958 for flowering time and seed size were identified. In order to identify a mutant line that is > 95% similar to ICC 4958 wild type at the genome level, 25 lines along with wild type were genotyped using 25 SSR markers that are equally distributed across the genome (Supplementary Table 1). Based on SSR marker data, a dendrogram (Supplementary Figure 1) was constructed using DARWin5 (Perrier et al., 2003). The mutant ICC4958-M3-2828 with > 95% similarity to the ICC 4958 wild type and phenotypically distinct for flowering time and seed size was selected for developing a MutMap population. A total of 28 F_1_ seeds were harvested by crossing ICC4958-M3-2828 and ICC 4958 wild type from July to September 2017 in the greenhouse at ICRISAT. During the crop season 2017–2018, F_1_s were advanced to F_2_ and a total of 204 F_2_ seeds were harvested.

### Phenotypic Diversity in MutMap Population and Preparation of Trait Bulks

A total of 204 F_2_ plants were phenotyped for early flowering and 100-seed weight during the crop season 2018–2019 in the field. The MutMap population had high phenotypic variability for both flowering time and seed size (Supplementary Table 2 and Supplementary Figures 2A,B). A negative correlation (*R* = −0.13) was observed among these traits (Supplementary Figure 2C). DNA from 15 F_2_ progeny that displayed early flowering (27–34 days, as EF pool) and late flowering (59–60 days, as LF pool) was combined. Similarly, we also combined DNA from 15 F_2_ progeny that displayed high 100-seed weight (43.0–46.2 g, as HSDW pool) and low 100-seed weight (27.0–41.0 gm, as LSDW pool) and subjected them to whole-genome sequencing using the Illumina HiSeq2500 platform (Supplementary Table 3).

### Whole-Genome Sequencing and Alignment of Short Reads

Both wild-type ICC 4958 and four trait bulks were sequenced to ∼10X of genome coverage. For each trait detail on the number of raw reads, filtered reads, mapped reads, average coverage, and average quality are presented in Table 1. In the case of flowering time-related pools, we obtained 44,907,030 and 37,699,572 cleaned bases for EF pool and LF pool, respectively. Similarly, for seed size-related pools, 43,818,340 and 50,125,538 cleaned bases were obtained for the HSDW pool and LSDW pool, respectively (Table 1). On aligning 47.41 million filtered reads to the CDC Frontier reference genome, 31.41 million reads were mapped and 332,395 SNPs were called. These SNPs were used to develop a consensus reference genome sequence for ICC 4958 by replacing them in the particular positions of CDC Frontier. The filtered bulk sequenced paired-end reads were aligned and SNPs were called against this reference assembly that yielded alignment results as follows: 27.12 M reads for the LF pool, 31.57 M reads for the EF pool, 35.30 M reads for the HSDW pool, and 30.81 M reads for the LSDW pool (Table 1).

**TABLE 1:** Summary of data generated and aligned on wild and mutant pools.

**Wild/mutant pools**	**Raw reads**	**Filtered reads**	**Number of reads mapped**	**Average coverage (X)**	**Average mapping quality (%)**
ICC 4958 (wild)	63,541,248	47,414,372	31,418,911	9.28	42.51
Days to flowering (Low; LDF)	58,196,006	44,907,030	31,571,460	9.49	42.38
Days to flowering (High; HDF)	51,747,822	37,699,572	27,123,905	7.97	42.53
100-seed weight (Low; LSDW)	58,041,216	43,818,340	30,815,440	9.19	42.37
100-seed weight (High; HSDW)	65,129,934	50,125,538	35,307,049	10.59	42.55

### Trait-Associated Genes and SNPs

**FIGURE 1:**
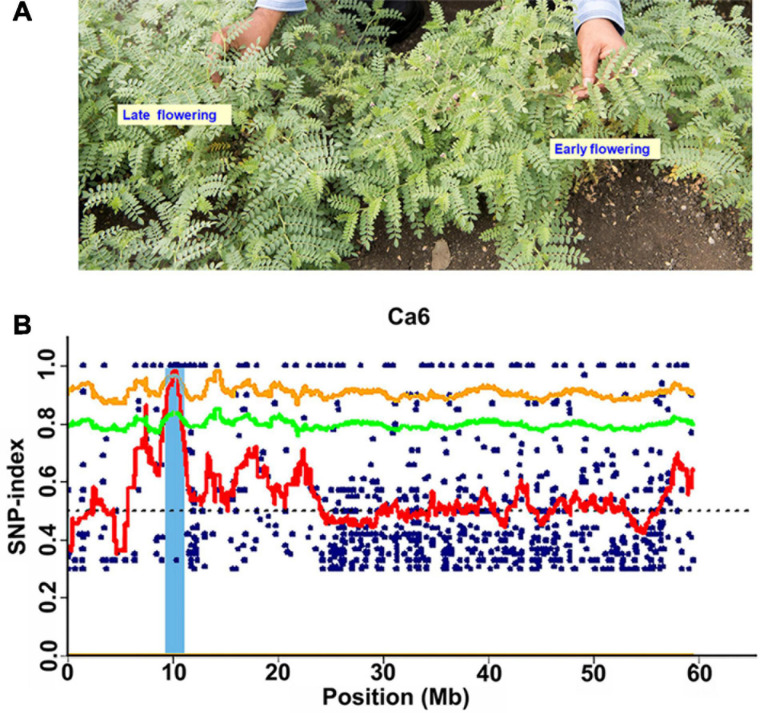
Phenotypic variation for flowering time and identification of candidate genomic region using MutMap approach. **(A)** Representative picture showing the variation in flowering in the MutMap population developed crossing ICC 4958-M3-2828 × ICC 4958 (wild). **(B)** A genomic region on Ca6 x-axis indicates the physical position of the chromosome, and the y-axis indicates the average SNP-index in a 2 Mb interval with a 10 kb sliding window.

**FIGURE 2:**
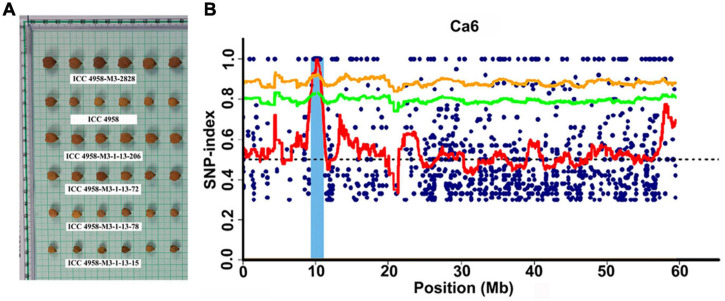
Phenotypic variation for seed size and identification of candidate genomic region using MutMap approach. **(A)** Representative picture showing the variation in seed size in the MutMap population developed crossing ICC 4958-M3-2828 × ICC 4958 (wild). **(B)** A genomic region on Ca6 x-axis indicates the physical position of the chromosome, and the y-axis indicates the average SNP-index in a 2 Mb interval with a 10 kb sliding window.

The Illumina short reads obtained for the four bulks were separately aligned to the reference sequence of ICC 4958 and then compared to the SNPs of each mutant bulk against wild-type ICC 4958 to identify the SNPs specific for each mutant bulk as well as their distribution on each pseudomolecule (Table 2). The SNP index for each SNP was also calculated. The number of SNPs among the bulks ranged from 3,993 (EF pool) to 5,771 (HSDW pool). In the case of EP pool flowering bulk, MutMap revealed 3,993 and 5,081 SNPs, of which 872 and 25 were candidate sites for the EF and LF pools, respectively, with a SNP index ≥ 0.8 (Table 2). While in the case of seed size bulks, MutMap revealed 4,777–5,771 SNPs, of which 771 and 1,078 were candidate sites for the LSDW and HSDW pools, respectively, with a SNP index ≥ 0.8 (Table 2). These SNPs were presumably the candidate mutations. However, it was not possible to pinpoint causal mutations from so many candidates. Further, for each bulk, SNP index vs. SNP genomic position graphs for the eight chickpea pseudomolecules were generated as shown in Supplementary Figures 3–6. The SNP index plots were very similar between the mutant and wild-type bulks across the entire genome. Nevertheless, a single genomic region on Ca6, between 9.76 and 12.96 Mb with a peak of SNP index 1, was identified in all four mutant bulks overlapping in this region that is missing from the wild-type bulk (Table 3). As expected, the SNP index was close to 0 across the genome, but within the unique genomic region identified on Ca6, between 9.76 and 12.96 Mb, its value was greater than zero. This was the only region that exhibited a SNP index difference of > 0 that is significant between the mutant and wild-type bulks. After identifying the region specific to mutant bulk, with a SNP index = 1, the SNPs therein (Ca6, between 9.76 and 12.96 Mb) were scrutinized in detail. Accordingly, we found a total of 38, 22,17,32 SNPs with a SNP index = 1 in the case of EF (Figure 1), LF, HSDW (Figure 2), and LSDW pools, respectively in the region for the mutant bulk (Table 3). Of the 102 candidate SNPs, 41 were unique candidate SNPs and 33 were found in more than one bulk. On annotation of the 74 SNPs (41 unique and 33 in more than one bulk), 48, 16, 7, and 3 were intergenic, intronic, synonymous, and missense SNPs, respectively (Supplementary Table 4). Among 22 SNPs with a SNP index = 1, 44 were CT, and eight were GA transitions in the case of LF pool (Supplementary Table 5). We identified 31 SNPs with a SNP index = 1 in the case of the EF pool, of which 17 were CT and 14 GA transitions (Supplementary Table 6). Among 31 SNPs with a SNP index 1, a SNP (Ca6_10099486) present in the gene Ca_08581 that encodes putative importin beta-3 (AtKPNB1), in a previous study upregulation of AtKPNB1 led to early flowering in Arabidopsis (Luo et al., 2013). Similarly, 17 and 32 mutations were identified with a SNP index = 1, in HSDW and LSDW pools, respectively (Supplementary Tables 7, 8). Further, among 22 SNPs, two SNPs are in the gene Ca_08530, which encodes aspartokinase homoserine dehydrogenase involved in the homoserine biosynthetic process, in the phosphorylation process, and in the oxidation–reduction process (Table 4).

**TABLE 2:** Identification and distribution of associated SNPs in the genome.

**Trait (bulk)**	**Number of SNPs**	**SNPs with a SNP index > 0.8**	**Pseudomolecules**							
			**Ca1**	**Ca2**	**Ca3**	**Ca4**	**Ca5**	**Ca6**	**Ca7**	**Ca8**
Days to flowering (Low)	5,081	872	864	503	486	856	626	860	735	151
Days to flowering (High)	3,993	25	667	385	395	648	508	705	556	129
100-seed weight (High)	5,771	1078	812	491	498	775	596	817	643	145
100-seed weight (Low)	4,777	771	1,068	566	552	960	653	999	794	179

**TABLE 3:** Summary of SNPs with SNP index 1 on chromosome Ca6.

**Trait**	**Region on Ca6 (Mb)**	**Total region (Mb)**	**SNPs with a SNP index = 1**
Days to flowering (Low)	9.77–12.96	3.19	31
Days to flowering (High)	9.77–11.09	1.32	22
100-seed weight (High)	9.76–10.68	0.92	17
100-seed weight (Low)	9.82–10.68	0.86	32

**TABLE 4:** Summary of candidate genes in the genomic region on chromosome Ca6 harboring candidate SNPs with SNP-index = 1.

**Reference/consensus base**	**SNP position**	**Gene**	**Sequence description**	**Biological process**	**Cellular component**	**Molecular function**
C/T	10,685,694	Ca_08529	Subtilisin-like protease SDD1-like protein	Proteolysis	–	Serine-type endopeptidase activity
C/T	10,670,959	Ca_08530	Aspartokinase-homoserine dehydrogenase	Homoserine biosynthetic process, phosphorylation, oxidation–reduction process	–	Aspartate kinase activity, homoserine dehydrogenase activity, NADP binding
C/T	10,678,606	Ca_08530	Aspartokinase-homoserine dehydrogenase	Homoserine biosynthetic process, phosphorylation, oxidation–reduction process	–	Aspartate kinase activity, homoserine dehydrogenase activity, NADP binding
G/A	10,509,893	Ca_08544	Probable magnesium transporter NIPA1	Magnesium ion transmembrane transport	Early endosome, plasma membrane, integral component of membrane	Magnesium ion transmembrane transporter activity
G/A	10,498,876	Ca_08547	Beta-galactosidase 9	Carbohydrate metabolic process	Vacuolar membrane, plant-type cell wall, integral component of membrane	Beta-galactosidase activity, carbohydrate binding
C/T	10,432,389	Ca_08552	TBC1 domain family member 13-like	Intracellular protein transport, activation of GTPase activity	Intracellular	GTPase activator activity, Rab GTPase binding
G/A	10,394,606	Ca_08553	UV radiation resistance-associated-like protein	Protein targeting to vacuole, SNARE complex assembly, multivesicular body sorting pathway	Endosome, cytosol, integral component of membrane	SNARE binding
G/A	10,259,516	Ca_08563	Mediator of RNA polymerase II transcription subunit 10b-like	Regulation of transcription by RNA polymerase II	Mediator complex	Transcription coregulator activity
G/A	10,226,669	Ca_08566	Serine/threonine-protein phosphatase PP2A catalytic subunit isoform X2	Protein dephosphorylation	–	Phosphoprotein phosphatase activity
C/T	10,190,456	Ca_08570	Acyl-activating enzyme 17, peroxisomal protein, putative	–	Integral component of membrane	Catalytic activity
G/A	10,099,486	Ca_08581	Importin beta-3, putative	NLS-bearing protein import into nucleus, ribosomal protein import into nucleus	Nuclear membrane, nuclear periphery	Nuclear localization sequence binding, Ran GTPase binding
G/A	10,040,897	Ca_08587	Acyl-activating enzyme 17, peroxisomal protein, putative	–	Integral component of membrane	Catalytic activity
C/T	9,828,083	Ca_08609	Hypothetical protein glysoja_010758	–	–	–
G/A	10,599,353	Ca_08537	S-Adenosyl-L-homocysteine hydrolase	One-carbon metabolic process, S-adenosylhomocysteine catabolic process	Cytosol	Adenosylhomocysteinase activity, NAD binding
G/A	10,498,876	Ca_08547	Beta-galactosidase 9	Carbohydrate metabolic process	Vacuolar membrane, plant-type cell wall, integral component of membrane	Beta-galactosidase activity, carbohydrate binding
G/A	10,259,516	Ca_08563	Mediator of RNA polymerase II transcription subunit 10b-like	Regulation of transcription by RNA polymerase II	Mediator complex	Transcription coregulator activity
C/T	10,190,456	Ca_08570	Acyl-activating enzyme 17, peroxisomal protein, putative	–	Integral component of membrane	Catalytic activity
G/A	10,099,486	Ca_08581	Importin beta-3, putative	NLS-bearing protein import into nucleus, ribosomal protein import into nucleus	Nuclear membrane, nuclear periphery	Nuclear localization sequence binding, Ran GTPase binding
G/A	10,040,897	Ca_08587	Acyl-activating enzyme 17, peroxisomal protein, putative	–	Integral component of membrane	Catalytic activity
C/T	10,025,005	Ca_08590	Receptor-like protein kinase	Protein phosphorylation	Integral component of membrane	Protein serine/threonine kinase activity, ATP binding
G/A	9,890,335	Ca_08601	LRR receptor-like kinase family protein	Protein phosphorylation	Integral component of membrane	Protein kinase activity, ATP binding
C/T	9,828,083	Ca_08609	Hypothetical protein glysoja_010758	–	–	–

### Markers for Early Flowering and Large Seed Size

**FIGURE 3:**
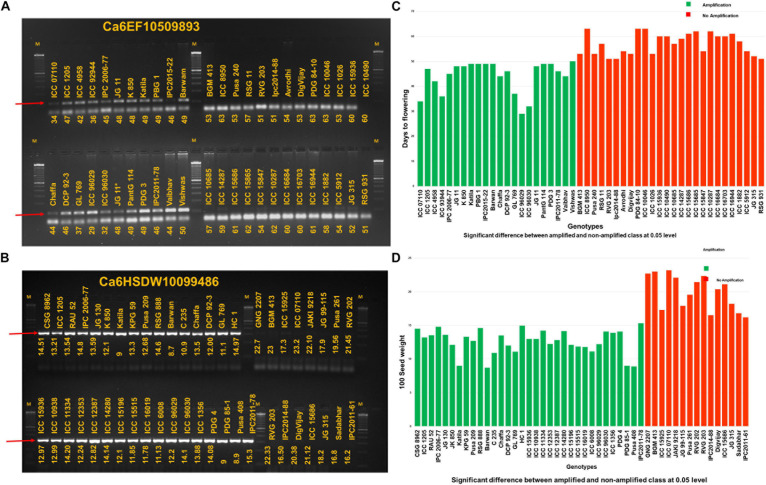
Markers developed for early flowering and seed size **(A)** The marker allele-specific marker Ca6EF10509893 for early flowering shows amplification in early flowering genotypes and no amplification in late flowering genotypes and **(B)** similarly, in the case of allele specific marker Ca6HSDW10099486 for 100-seed weight amplification can be seen and no amplification in case of genotypes with high 100-seed weight. M is the 100-bp marker. Allele-specific amplicons are indicated with the red color arrow. A clear significant difference (*p* < 0.05) between the amplified and non-amplified genotypes based on their phenotypic values for Ca6EF10509893 and Ca6HSDW10099486 can be visualized in **(C,D)**, respectively.

Of 102 candidate SNPs on Ca6, between 9.76 and 12.96 Mb, 74 candidate SNPs were targeted to design primer pairs using WASP (see text footnote 1). A total of 12 allele-specific primer pairs were designed, and the primer sequence information is provided in Supplementary Table 9. Twelve primer pairs were initially amplified on a set of eight chickpea genotypes. Of twelve primer pairs, seven primer pairs had amplification on all eight genotypes tested. However, allele-specific amplification was obtained for Ca6HSDW10099486, Ca6HSDW9890335, Ca6HSDW9828083, and Ca6EF10509893. Hence, these 4 markers were validated on 82 select chickpea germplasm lines (47 for early flowering and 48 for seed size). As a result, one marker each for EF (Ca6EF10509893; Figure 3A) and HSDW (Ca6HSDW10099486; Figure 3B), with allele-specific amplification and high accuracy in the tested germplasm lines, has the potential to be used for improving the early flowering and seed size in chickpea. A clear significant difference (*p* < 0.05) between the amplified and non-amplified genotypes based on their phenotypic values for Ca6EF10509893 and Ca6HSDW10099486 can be visualized in Figures 3C,D, respectively. Nevertheless, these markers need to be tested on large germplasm sets for their efficiency before being used in early-generation selection in chickpea breeding programs.

## Discussion

Early flowering and seed size are the two important traits in chickpea as short-duration cultivars can escape terminal drought and high/low-temperature stresses, and enhanced seed size increases the yield to compensate for yield loss due to drought stress. Although early flowering accessions of desi and kabuli types have been identified from germplasm collections (Upadhyaya et al., 2007) and super-early lines (ICCV 96029 and ICCV 96030) have been developed, there is a need for the identification of candidate genes and causal SNPs to accelerate the development of climate-resilient chickpea varieties. In the past, early flowering genes and their allelic relationships were reported based on the understanding of trait genetics (Gaur et al., 2014). Major QTLs for 100-seed weight were reported and were also fine-mapped (Varshney et al., 2014c; Jaganathan et al., 2015; Kale et al., 2015). However, none of the studies focused on the identification of candidate genes and causal SNPs responsible for flowering time and seed size.

The development of climate-resilient cultivars will make small-holder agriculture profitable in the anticipated climate change scenarios. In addition to the integration of multidisciplinary approaches in breeding, the adoption of a 5Gs breeding approach will accelerate genetic gains as well as meet the future demands of nutritious food (Varshney et al., 2018, 2020). In the case of legumes, sequence-based breeding in the post-genome sequence era has improved the efficiency of developing climate-resilient cultivars considerably (Varshney et al., 2019a). In this study, we report the identification of genes and SNPs using a MutMap approach, as well as the development of markers for use in chickpea breeding programs toward the development of cultivars with early flowering and large seed size.

We developed a MutMap population by crossing ICC 4958-M3-2828 to ICC 4958 (wild type) to identify the candidate genes and causal SNPs for early flowering and large seed size. A previous MutMap approach and its variants were successfully deployed to localize the position of genes for agronomically important genes in cereals such as rice (Abe et al., 2012; Hu et al., 2016; Deng et al., 2017; Yuan et al., 2017; Nakata et al., 2018), sorghum (Jiao et al., 2018), and also in cucumber (Xu et al., 2018). This is the first study deploying a MutMap approach in chickpea. In this study, we demonstrated that MutMap is a powerful approach to identify causal homozygous mutation in bulked F_2_ plants selected for a phenotype of interest. Although the MutMap method was initially considered for mapping of monogenic recessive (mutations) gene-controlled traits, it is now possible to map dominant mutations through progeny testing and bulking of homozygous dominant F_2_ individuals. In total 3,32,395 SNPs were used to develop a consensus reference genome sequence by replacing them at a particular position on the CDC Frontier genome. The sliding window analysis was applied to identify the trait linked SNPs with a SNP index value = 1. The extreme bulks sequenced reads were aligned to a consensus reference genome through a sliding window approach (moving averages). The SNPs in the sequenced F_2_ population were in heterozygous state, show a 1:1 segregation pattern, 50% SNPs were mutant type, and the remaining 50% SNPs were wild type represented by a SNP index value of 0.5. If the F_2_ population SNPs are in homozygous condition, then these are linked to the mutant phenotype (100% mutant reads, 0% wild-type reads represented as the SNP index value = 1). A SNP index value of 1 or near to 1 indicates the causal mutant SNP linked to the trait of interest, whereas a value of 0.5 indicates SNP not linked to the trait. The SNPs possess a SNP index value of 1 or near to 1 can be successfully targeted for marker development that can potentially be used in breeding. The number of SNPs among the bulks ranged from 3,993 (EF pool) to 5,771 (HSDW pool). Nevertheless, we identified only 102 candidate SNPs with a SNP index = 1. Interestingly, a genomic region harboring the candidate SNPs for all four bulks was on Ca6. In previous reports on chickpea, major QTLs for flowering time were reported on CaLG04 in the genomic region referred to as “*QTL-hotspot.*” Nevertheless, major QTLs corresponding to flowering time genes *efl-1* from ICCV 96029, *efl-3* from BGD 132, and *efl-4* from ICC 16641 were mapped on CaLG04, CaLG08, and CaLG06, respectively (Mallikarjuna et al., 2017). This indicates that flowering is a complex process coordinated by environmental and endogenous factors to ensure plant reproduction in appropriate conditions (Kumar et al., 2016). The “*QTL-hotspot*” was reported on Ca4 from 9.1 to 16.1 Mb (Varshney et al., 2014a). The relative positions of *efl1* and *efl2* genes mapped by Mallikarjuna et al. (2017) to “*QTL-hotspot*” were determined using the primer sequence of flanking markers NCPGR21 and GAA47 using *blastn -task “blastn-short*.” The marker for *efl1* (NCPGR21) was found to be present inside the “*QTL-hotspot*” at ∼10 Mb on Ca4, whereas the marker for *efl2* (GAA47) was found present ∼818.1kb upstream of the “*QTL-hotspot*” on the genome at ∼8.3 Mb on Ca4. Further, the flowering time genes are distributed throughout the genome and are dependent on the genetic background. Genome-wide distribution of flowering time genes is not uncommon and was recently also reported in cucurbits (Yi et al., 2020).

On annotating the candidate SNPs in this genome region on Ca6, we identified that these SNPs are located within candidate genes that are involved in flowering time as well as in seed development. For instance, the candidate SNPs, Ca_10137361 and Ca6_11657245, present in Ca_08579 and Ca_25060 genes, respectively, are associated with calmodulin sensing Ca^2+^ signals and are reported to be involved in flowering time (Kumar et al., 2016). The SNP Ca6_10099486, present in gene Ca_08581 that encodes putative importin beta-3, was reported to play a key role in drought tolerance in *Solanum tuberosum* (Xu et al., 2020). AtKPNB1, which is a member of the Arabidopsis importin family, was reported to be a gene associated with ABA sensitivity at germination, early seedling development, drought tolerance, and stomatal closure regulation; it is expressed in various organs and any specific tissues in listed organs such as leaves, roots, and flowers (Luo et al., 2013). Further, a SNP Ca6_10685694, present in gene Ca_08529, encodes for subtilisin-like protease SDD1 (STOMATAL DENSITY AND DISTRIBUTION-1) and SDD1-like transcripts in *Solanum chilense* and *Solanum lycopersicum*. SDD1 is also known to play an important role in early leaf and flower development in both tomato species (Morales-Navarro et al., 2018). Similarly, a SNP Ca6_10498876, present in gene Ca_08547 that encodes beta-galactosidase9 to be expressed during fruit ripening, plays a major role in abscission, early onset of growth, and development processes in flowers and fruitlets (Wu and Burns, 2004). The genes Ca_08587 and Ca_08570 encode for the acyl-activating enzyme 17 (AAE17) reported to having a functional role in seed development. The Ca_08530 gene encodes aspartokinase-homoserine dehydrogenase (AK/HSD) enzyme involved in aspartate kinase activity, homoserine dehydrogenase activity, and NADP binding activity. AK/HSD-GUS gene has been reported to be expressed in actively growing tissues and seed development. A SNP Ca6_10685694, present in the gene Ca_08529 located on Ca6, encodes subtilisin-like protease (SBT) SDD1-like protein. SBTs have been shown to control diverse developmental processes like stomatal distribution and density (Berger and Altmann, 2000; Von Groll et al., 2002). Two allele-specific markers, Ca6EF10509893 for early flowering and Ca6HSDW10099486 for 100-seed weight, developed in this study were also validated on a select set of chickpea germplasm lines. These markers can be further tested on a larger germplasm panel with the potential to be converted to high-throughput assays for early-generation selection in chickpea breeding programs.

## Conclusion

MutMap has the advantage of both bulk segregant analysis and WGRS (whole-genome resequencing) approaches and enables the identification of candidate genes and causal SNPs. In the present study, we report 102 candidate SNPs in 22 candidate genes. The candidate genes identified in this study are involved in early flowering as well as enhanced seed size. Further, we also report the development and validation of markers for use with chickpea. Testing of these markers on a large and diverse panel of genotypes will be required prior to use in breeding programs for improving these traits.

## Data Availability Statement

The datasets presented in this study can be found at: https://www.ncbi.nlm.nih.gov/bioproject/PRJNA715624.

## Author Contributions

MT conceived the project and secured funding. PM performed the experiments and prepared the first draft. BPM developed the MutMap population. DK, PB, and AWK performed the bioinformatics analysis. RP performed the experiments and phenotyped the population. UCJ contributed germplasm for marker validation. RKV, AC, and PS contributed the resources. MT and RKV wrote the review, edited manuscript and finalized the manuscript. All authors read and approved the manuscript.

## Conflict of Interest

The authors declare that the research was conducted in the absence of any commercial or financial relationships that could be construed as a potential conflict of interest.

## Footnotes

1 http://bioinfo.biotec.or.th/WASP

## Supplementary Material

Supplementary Figure 1 Dendrogram constructed based on the 25 SSR markers distributed across chickpea genome indicates more than 95% similarity between ICC 4958 (wild type) and ICC 4958-M3-2828.

Supplementary Figure 2 Frequency distribution and correlation among days to flowering and 100-seed weight. Histogram showing the frequency distribution of days to flowering **(A)** and 100-seed weight **(B)**. A negative correlation of 0.13 was observed between these traits.

Supplementary Figure 3 Single nucleotide polymorphism (SNP) index plots for all eight pseudomolecules for early flowering pool. The red line indicates the sliding window average of 2-Mb interval with an increment of 10 kb for SNP index.

Supplementary Figure 4 Single nucleotide polymorphism index plots for all eight pseudomolecules for late flowering pool. The red line indicates the sliding window average of 2 Mb interval with an increment of 10 kb for SNP index.

Supplementary Figure 5 Single nucleotide polymorphism index plots for all eight pseudomolecules for large seed size pool. The red line indicates the sliding window average of 2 Mb interval with an increment of 10 kb for SNP index.

Supplementary Figure 6 Single nucleotide polymorphism index plots for all eight pseudomolecules for small seed size pool. The red line indicates the sliding window average of 2 Mb interval with an increment of 10 kb for the SNP index.

Supplementary Table 1 Allelic data of 25 SSR markers distributed on the genome generated on mutant and wild lines.

Supplementary Table 2 Phenotyping data generated on MutMap population derived from ICC 4958-M3-2828 × ICC 4958.

Supplementary Table 3 Summary of the extreme bulks prepared for flowering time and seed size mutant based on phenotyping data generated on MutMap population derived from ICC 4958-M3-2828 × ICC 4958.

Supplementary Table 4 Summary of non-redundant candidate SNPs in the genomic region of Ca6 and their annotations.

Supplementary Table 5 Summary of SNPs identified in late flowering pool.

Supplementary Table 6 Summary of SNPs identified in early flowering pool.

Supplementary Table 7 Summary of SNPs identified in large seed size pool.

Supplementary Table 8 Summary of SNPs identified in small seed size pool.

Supplementary Table 9 Details of primer pairs designed for candidate SNPs.
