# Supplementary figures and images for: MutMap Approach Enables Rapid Identification of Candidate Genes and Development of Markers Associated With Early Flowering and Enhanced Seed Size in Chickpea (Cicer arietinum L.)
Source: Front Plant Sci. 2021 Jul 12;12:688694. doi: 10.3389/fpls.2021.688694 (PMC8313520; doi:10.3389/fpls.2021.688694)

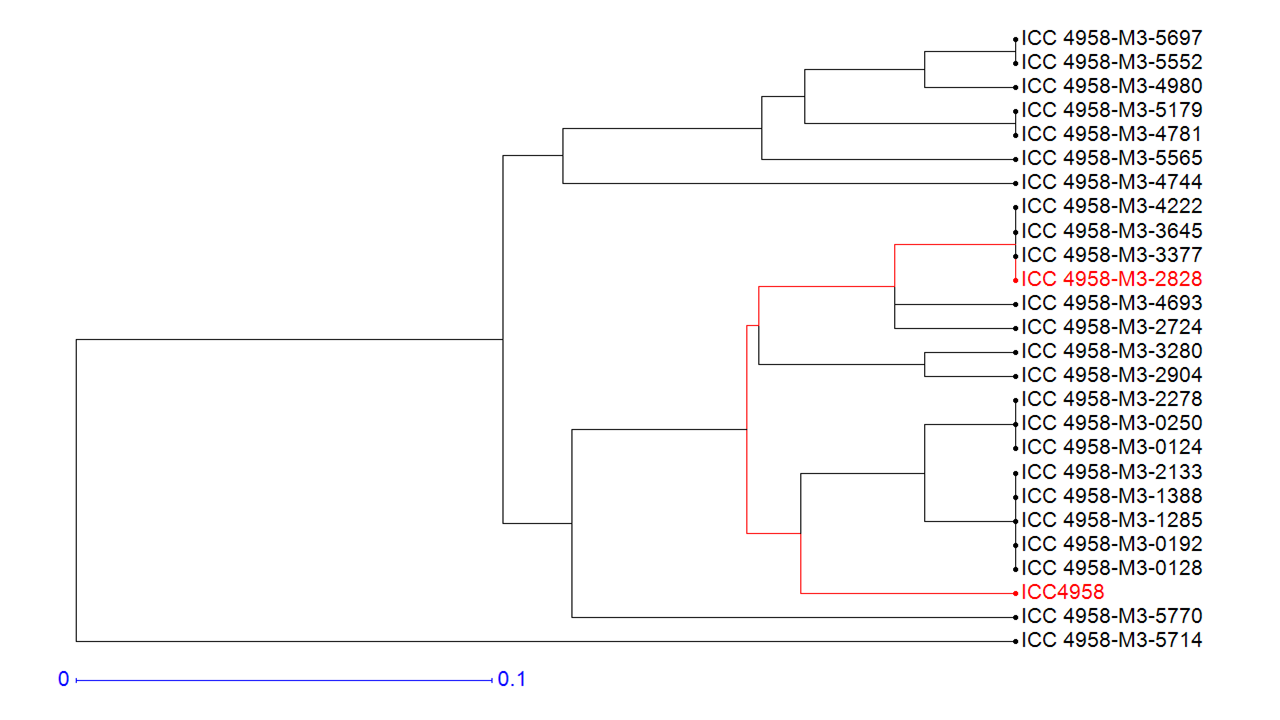

Supplement: Supplementary Figure 1 — Dendrogram constructed based on the 25 SSR markers distributed across chickpea genome indicates more than 95% similarity between ICC 4958 (wild type) and ICC 4958-M3-2828. [file Image_1.TIF]

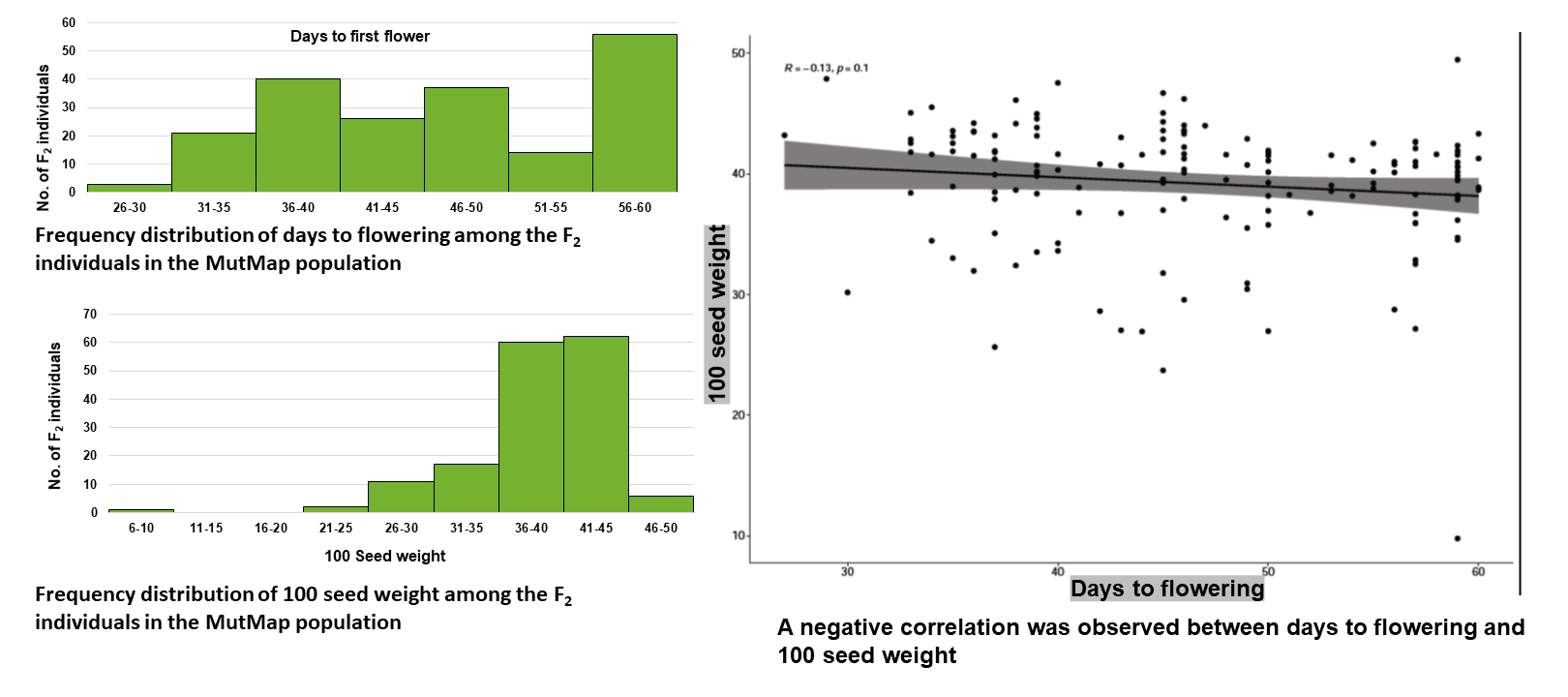

Supplement: Supplementary Figure 2 — Frequency distribution and correlation among days to flowering and 100-seed weight. Histogram showing the frequency distribution of days to flowering (A) and 100-seed weight (B). A negative correlation of 0.13 was observed between these traits. [file Image_2.TIF]

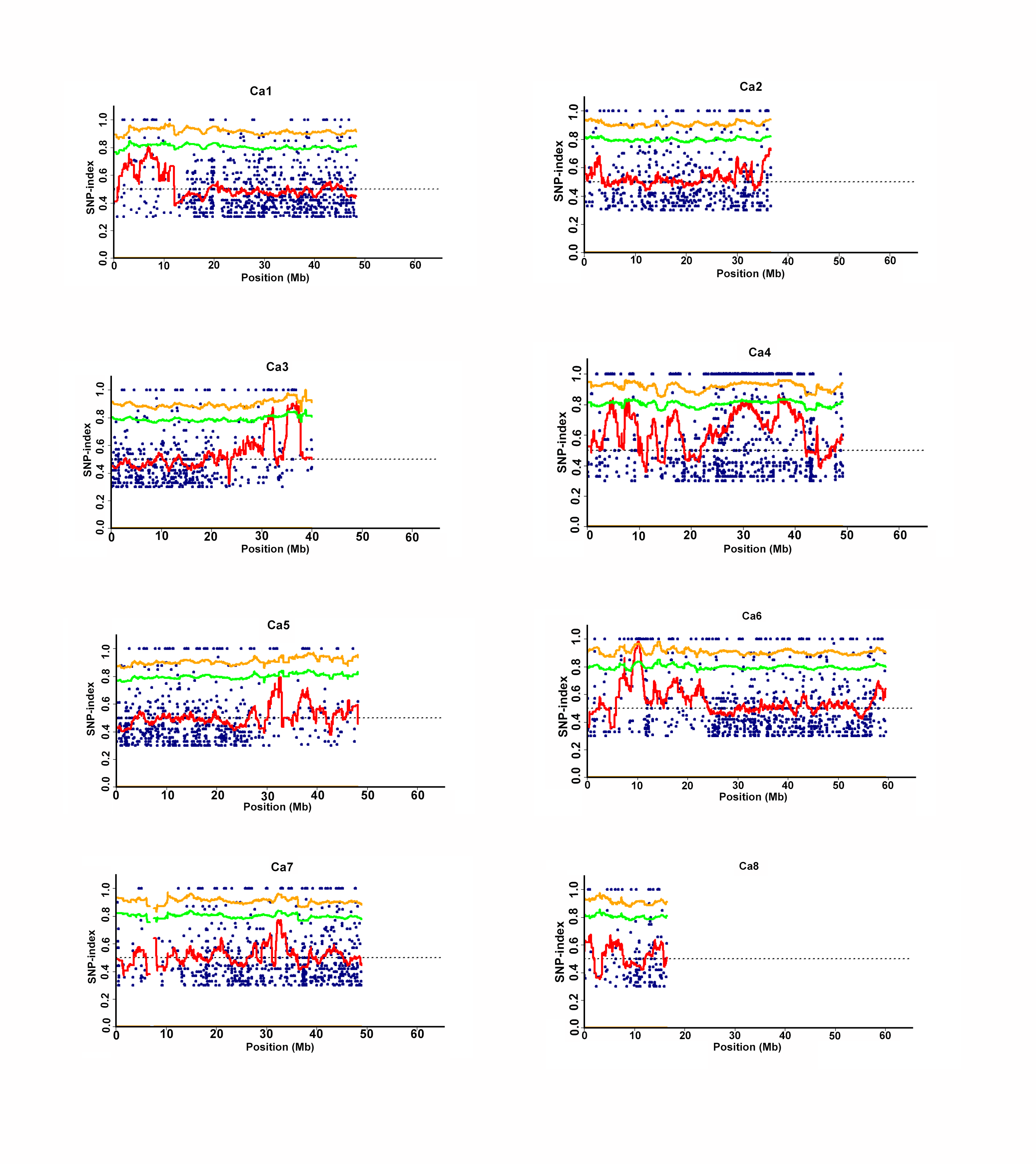

Supplement: Supplementary Figure 3 — Single nucleotide polymorphism (SNP) index plots for all eight pseudomolecules for early flowering pool. The red line indicates the sliding window average of 2-Mb interval with an increment of 10 kb for SNP index. [file Image_3.TIF]

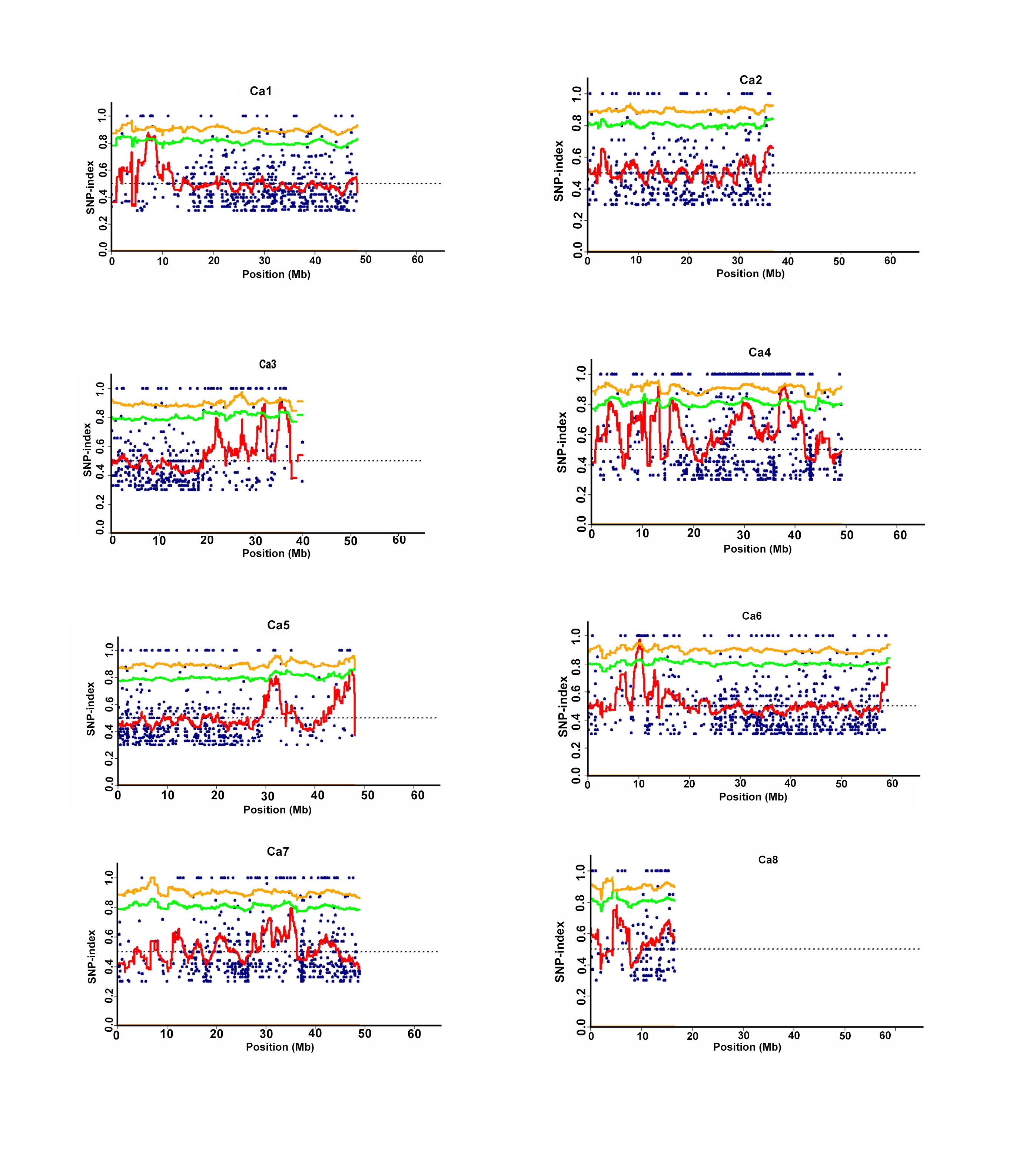

Supplement: Supplementary Figure 4 — Single nucleotide polymorphism index plots for all eight pseudomolecules for late flowering pool. The red line indicates the sliding window average of 2 Mb interval with an increment of 10 kb for SNP index. [file Image_4.TIF]

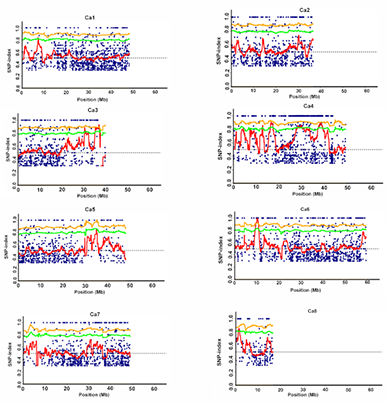

Supplement: Supplementary Figure 5 — Single nucleotide polymorphism index plots for all eight pseudomolecules for large seed size pool. The red line indicates the sliding window average of 2 Mb interval with an increment of 10 kb for SNP index. [file Image_5.tif]

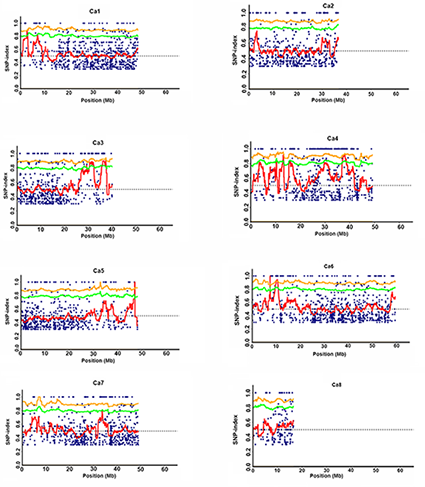

Supplement: Supplementary Figure 6 — Single nucleotide polymorphism index plots for all eight pseudomolecules for small seed size pool. The red line indicates the sliding window average of 2 Mb interval with an increment of 10 kb for the SNP index. [file Image_6.tif]
